# Supplementary material for: Siraitia grosvenorii Extract Attenuates Airway Inflammation in a Murine Model of Chronic Obstructive Pulmonary Disease Induced by Cigarette Smoke and Lipopolysaccharide
Source: Nutrients. 2023 Jan 16;15(2):468. doi: 10.3390/nu15020468 (PMC9865488; doi:10.3390/nu15020468)
Supplement: Supplementary file 1 [file nutrients-15-00468-s001.zip › nutrients-2114850-supplementary.pdf]

Supplementary Figure S1. Raw data of western blots for Figure 2.

A

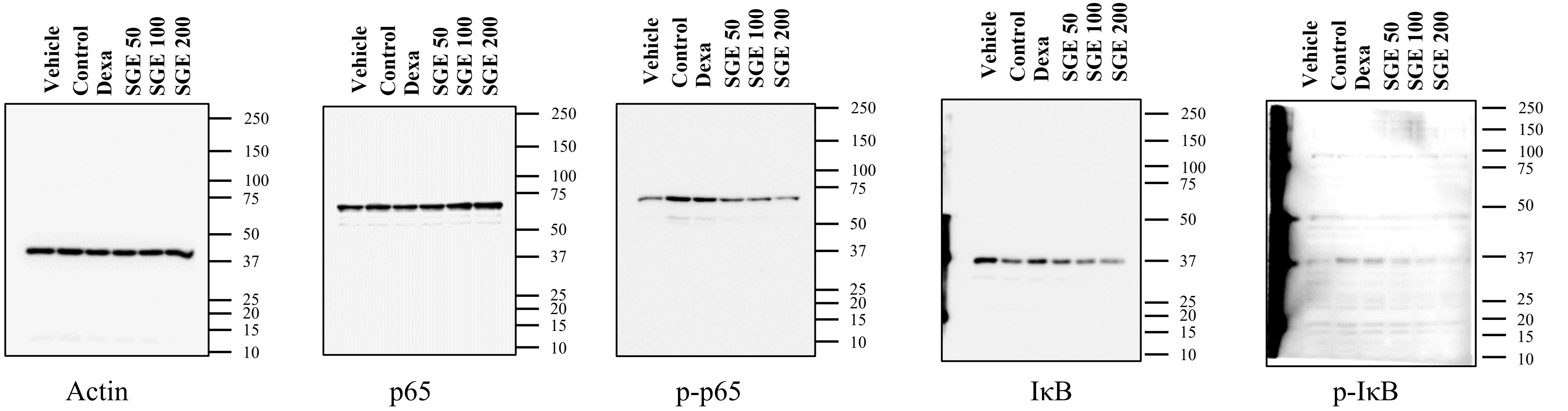

B

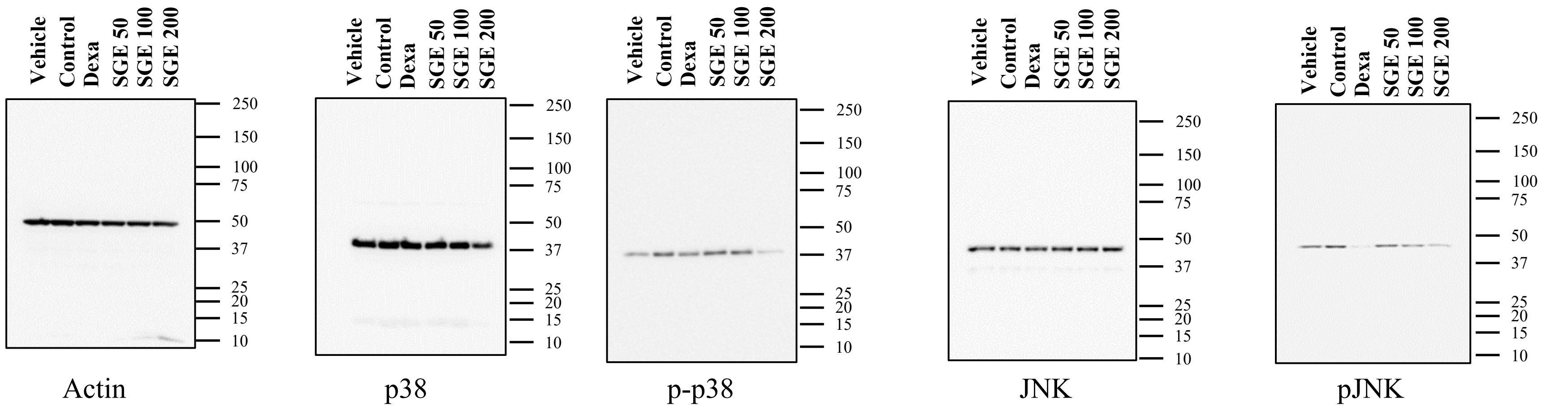

Supplementary Table S1. Primers sequence used in qRT-PCR analysis.

Gene	Primer	Oligonucleotide Sequence (5'-3')
hActin	F	GCGGGAAATCGTGCGTGACA
	R	GATGGAGTTGAAGGTAGTTT
hIL-1β	F	ACGCTCCGGGACTCACAGCA
	R	TGAGGCCCAAGGCCACAGGT
hIL-6	F	AGAGTAGAGGAACAAGCC
	R	TACATTTGCCGAAGAGCCCT
hIL-8	F	CTCTTGGCAGCCTTCCTGATT
	R	ACTCTCAATCACTCTCAGTTC
hIL-17	F	TCAACCCGATTGTCCACCAT
	R	GAGTTTAGTCCGAAATGAGGC
hCXCL1	F	GAAAGCTGCCTCAATCCTG
	R	CTTCCTCCTCCCTTCTGGTC
hTNF-α	F	GCCCAGGCAGTCAGATCATCT
	R	TTGAGGGTTTGCTACAACATG
hIRAK1	F	TCAGCTTTGGGGTGGTAGTG
	R	TAGATCTGCATGGCGATGGG
mActin	F	TGGAATCCTGTGGCATCCAT
	R	TAAAACGCAGCTCGTAACAG
mTNF-α	F	CCTGTAGCCCACGTCGTAGC
	R	TTGACCTCAGCGCTGAGTTG
mMIP-2	F	ATGCCTGAAGACCCTGCCAAG
	R	GGTCAGTTAGCCTTGCCTTTG
mCXCL-1	F	CCGAAGTCATAGCCACAC
	R	GTGCCATCAGAGCAGTCT
mMUC5AC	F	AGAATATCTTTCAGGACCCCT
	R	ACACCAGTGCTGAGCATACTT
mTRPV1	F	CATCTTCACCACGGCTGCTTAC
	R	CAGACAGGATCTCTCCAGTGAC
mTRPA1	F	TGAGATCGACCGGAGT
	R	TGCTGAAGGCATCTTG
